# Review of Breast Conservation Therapy: Then and Now

**DOI:** 10.5402/2011/617593

**Published:** 2011-09-12

**Authors:** Susan Hoover, Elizabeth Bloom, Sunil Patel

**Affiliations:** ^1^Department of Surgical Oncology, The University of Texas MD Anderson Cancer Center, Houston, TX 77030, USA; ^2^Division of Radiation Oncology, The University of Texas MD Anderson Cancer Center, Houston, TX 77030, USA; ^3^Department of General Oncology, The University of Texas MD Anderson Cancer Center, Houston, TX 77030, USA

## Abstract

Breast conservation therapy (BCT), which is the marriage of breast conserving surgery and radiation therapy to the breast, has revolutionized the treatment of breast cancer over the last few decades. Surgical direction had seen a heightened interest in the performance of cosmetically superior partial and segmental resections in breast conservation as well as increased demand by patients for breast preservation. The broadening of approaches to delivery of breast irradiation from whole breast to accelerated partial breast has allowed more patients to opt for breast conservation and allowed for what appears to be comparable measurable outcomes in emerging data. As well, the addition of state-of-the-art chemotherapeutic and hormonal therapies has allowed improved outcomes of patients from both local regional recurrence and overall survival standpoints. This paper will provide an overview of BCT and review some of the newest developments in optimizing this therapy for patients with breast cancer from a surgical-, medical-, and radiation-oncology standpoint.

## 1. Surgical Perspective

### 1.1. Introduction

**Figure 1:**
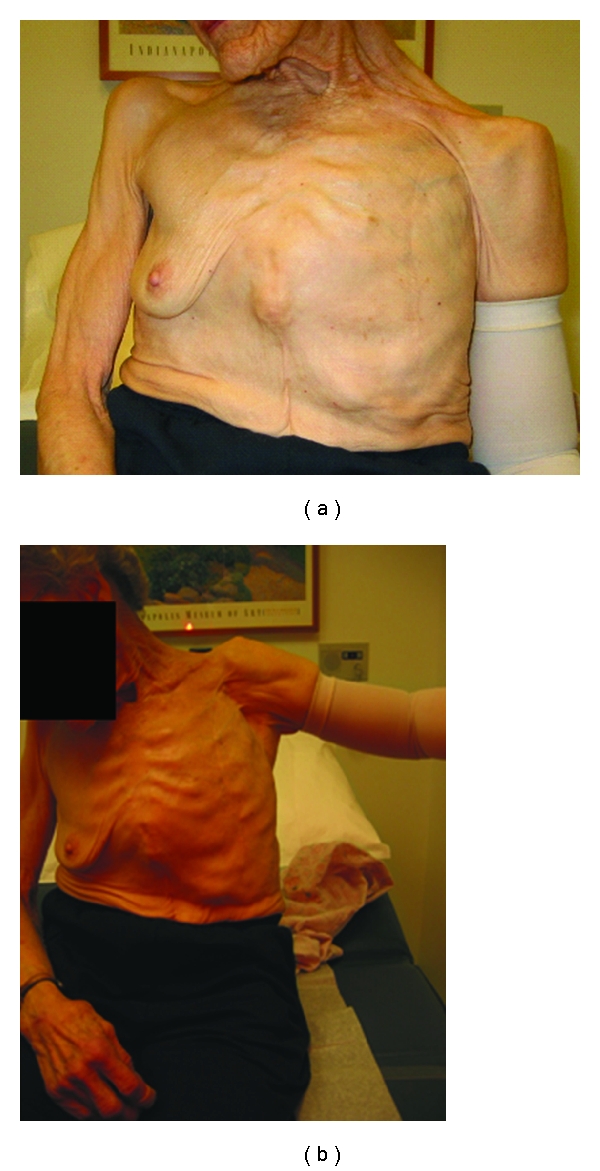
This patient serves as a reminder of the physical deformity (a) and morbidity associated with the Halstedian radical mastectomy. Note the compression sleeve for control of lymphedema and the decreased range of motion of the patient's left arm (b).

**Figure 2:**
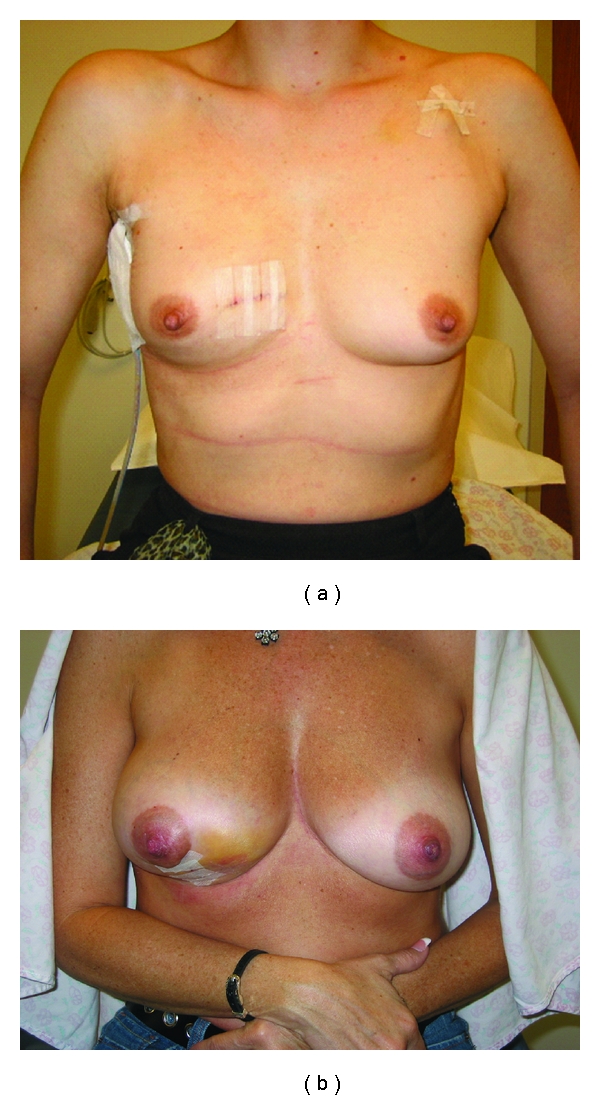
These patients are examples of the retention of body image with breast conservation surgery.

Much has changed in the management of breast cancer especially over the last few decades. The shift from the Halstedian radical mastectomy (Figure 1) to BCT serves as a remarkable example of the advances in surgical care of the breast cancer patient. The Breast conservation has improved the quality of life for many patients in terms of retention of body image and overall decreased physical morbidity (Figure 2). The Data also support that BCT affords patients' the same overall survival without statistically significant increased local recurrence rates [1–5]. The continued success of BCT has evolved from advances in surgical techniques and pathologic analyses with the application of state-of-the-art radiation and chemotherapeutic regimens.

### 1.2. Brief History of Surgery for Breast Cancer

A brief tour through the history of surgical therapy of breast cancer is warranted to better put into context the treatment of breast cancer as we know it today. In the late 19th century, Halstead popularized the radical mastectomy [6, 7]. This surgical approach involved *en bloc* resection of the affected breast, overlying skin, pectoral muscles, and axillary lymphadenectomy. At the time, this extensive procedure was performed to gain local-regional control of disease that was thought to primarily spread in a stepwise fashion from the breast through lymphatics to the lymph nodes, where it remained latent before spreading to distant organs. The patients of this time typically presented with locally advanced disease or neglected disease that in many respects justified such a radical approach. Ironically, women of the time, knowing what awaited them surgically should they be diagnosed with breast cancer, might be known to delay seeking medical care, laying the ground work for a surgical self-fulfilling prophecy of sorts [8]. 

Geoffrey Keynes reported in the early 20th century equal results of local breast tumor excision with radium irradiation to traditional Halstedian radical mastectomy (RM) [8]. Despite this, the practice of RM continued for decades to follow. With time, the RM approach fell out of favor as it became recognized that patient-treatment failure was due to systemic spread of disease prior to surgery and not due to inadequate surgery. The emergence of the modified radical mastectomy (MRM), which involves removal of the breast and typically Level I and II nodal basins, was then seen. More and more patients by the 1970s were presenting with smaller tumors not fixed to the underlying pectoral muscle, which made adoption of MRM a natural choice. As well, both retrospective and prospective studies of this time period revealed no difference between MRM and RM in terms of patient survival [9–12]. 

Following this, multiple prospective randomized trials demonstrated that patient survival after undergoing BCT (consisting of local tumor excision, axillary dissection, and whole breast irradiation) was equivalent to mastectomy in the treatment of invasive breast cancer [1–5]. 

With the advent of sentinel lymph node biopsy (SLNB), more patients are undergoing BCT or mastectomy with this targeted axillary lymph node sampling, which has decreased patient morbidity in terms of range of motion of the arm and lymphedema. Three-year followup data from the NSABP B-32 study concluded a superiority of SLNB to axillary nodal dissection when it came to postsurgical morbidity outcomes [13].

### 1.3. Breast Conservation Surgery

For patients to benefit from BCT, it must first be offered and delivered to them by the surgeon. Despite the decades of work and research dedicated to establishing the equivalence of BCT to mastectomy in the treatment of breast cancer, there remain a disproportionately lower number of women undergoing BCT as compared to mastectomy for disease processes that are otherwise amenable to conservation techniques. In 1990, a National Institutes of Health Consensus Development Conference on the treatment of patients with early stage invasive breast cancer recommended breast conservation for the majority of women with Stage I and II diseases. Even after that conference, it was noted at the time that although BCT was performed more often than previously, there were still barriers that existed to the widespread adoption of the treatment [14]. Fast forward to more present time and there is still documented underutilization of BCT for appropriate candidates with numerous studies dissecting the possible reasons for this practice [15–20]. 

In assessing a patient for candidacy for breast conservation, there are psychosocial aspects that need to be considered as well as medical aspects. From a psychosocial aspect, there is no question that counseling of patients to their options is a time-intensive endeavor and can be very emotionally charged as the patient is faced not only with a new diagnosis of cancer but is faced with multiple decision trees that ultimately affect her body image, her thoughts on her own mortality, and her social-, work-, and family-life. From a medical perspective, patients that are best suited for BCT are those with solitary, primary breast lesions whose surgery can achieve negative microscopic margins, and are medically suitable for chest wall irradiation. When considering the patient's case for BCT versus mastectomy, the practitioner must take into account tumor to breast size ratio, multicentricity, tumor characteristics such as extensive mammographic calcifications, technical ability to clear margins, and any contraindications for radiation therapy [21]. For example, if a patient has a relatively small breast size with a large area of microcalcifications, she may not prove to be the best candidate for conservation, lest inadequate margin clearance or poor cosmetic outcome results. However, in cases where the patient has a relatively small breast size in relation to a tumor mass, neoadjuvant chemotherapy may provide an avenue for tumor size reduction and ultimate successful breast conservation surgery. In general, the performance of breast conservation therapy in early stage disease is not limited by tumor size (except as noted above), tumor type, nodal status, or biologic characteristics of the tumor. Even with this latitude for effective surgery, there still exists some surgeon bias as noted by Keating et al. where only 71% of women reported that their surgeon discussed both BCT and mastectomy with them [22]. Others have reported surgeon bias as a driving force to the performance of mastectomy to BCT. One study surveyed surgeons regarding the management of T1 tumors and 56% of the group indicated that they did not believe mastectomy and BCT were equal treatment options or that they biased their discussions with patients in favor of mastectomy [23]. Morrow et al. showed that medical contraindications to BCT were not a major factor in the execution of BCT in their institution. They implemented a multidisciplinary approach to patient management with a standard set of criteria for BCT eligibility, which they felt minimized confusion about which patients were candidates for BCT. This points more to other potential factors such as surgeon bias as influencing patient choice of BCT versus mastectomy. They suggest that changes in surgeon attitude and continuing education about patient selection may do more to increase the utilization of BCT [24].

Simplistically put, breast conservation therapy offers patients retention of body habitus most closely to the natural state, although surgical alterations of the breast, especially with the addition of radiation, can often lead to suboptimal cosmetic outcomes [25]. Wang et al. reported that 28% of BCT in their series were dissatisfied with their cosmetic outcome and this group was more likely to have a negative change in their body image when compared with patients who were satisfied with their cosmetic result. They also reported that age younger than 52 years and the resection from the upper inner quadrant from the breast were risk factors predicting patient dissatisfaction [26].

Although there are many patients who experience good, primary cosmetic outcomes with BCT, there is emerging literature on the concept of oncoplastic breast conservation surgery for patients with suboptimal cosmetic outcomes. This concept has been widely published on in the last few years [27–30] and involves a one-stage combined approach by the oncologic and reconstructive surgeons, whereby reconstruction of the conserved breast is performed with a volume-displacing procedure involving local tissue reshaping and rearrangement by reduction mammoplasty or mastopexy to fill in the dead space created by tumor resection [27]. Meretoja et al. reported on their small series of 15 patients undergoing oncoplastic surgery post-breast conservation where 84% of patients had negative margins and acceptable cosmetic results[29]. In a larger series by Bong et al., the authors report on 167 patients who underwent oncoplastic mastopexy reconstruction after breast conservation surgery. In their series, they demonstrated the utility and adequacy of oncoplastic techniques when addressing larger or difficultly positioned tumors for breast conservation. They showed that margin positivity could be addressed easily by directed, single-face excision without substantially affecting cosmesis [31].

Despite the challenges that exist with the broader application of BCT, it is likely that as patients are more and more exposed to the information highway and have access to medical data at their fingertips, their demand for optimal surgical management will increase. And as Melvin Silverstein sites in reference to oncoplastic techniques, which can be just as appropriately said about BCT, “it will soon become patient driven and demanded. If surgeons do not offer it, their patients will drift away to surgeons who do” [28].

## 2. Medical Oncology Perspective

### 2.1. Neoadjuvant Systemic Therapy

In treating operable and locally advanced breast cancer, the clinician hopes to achieve three primary aims:

1. Eradication of disease (cure),
2. Minimization of morbidity,
3. Acceptable cosmesis.

Increasing acceptance of neoadjuvant systemic therapy (NST) as a standard of care has evolved as trials have established both efficacy and increased breast conservation rates with this approach. Additional benefits, including the prognostic value of NST response and individualized NST approaches based on tumor characteristics are likely to alter care delivery in the near future.

Neoadjuvant therapy was initially described as a single modality (chemotherapy) for a limited indication (locally advanced or inflammatory tumors) [32]. Recent advances, detailed below, have broadened the definition of NST to include chemotherapy, endocrine therapy, and targeted therapy, and expanded the indications to include Stage I–III breast cancers.

### 2.2. Efficacy of NST

NST offers a number of theoretical advantages over conventional adjuvant therapy, and there are hypothetical disadvantages worth noting as well (Table 1). Hypotheses notwithstanding, now that a number of NST trials have been completed, some reliable paradigms have emerged (1) in terms of disease-free and overall survival, neoadjuvant chemotherapy is equivalent to traditional adjuvant chemotherapy, (2) NST results in higher rates of BCS as compared to identical postoperative treatment, (3) response to NST provides valuable prognostic information, and (4) NST provides an excellent platform to conduct novel biomarker-driven studies with the aim of advancing the standard of care.

Two landmark neoadjuvant chemotherapy (NCT) trials recently reported long-term followup data. EORTC 10902 and NSABP B-18 randomized patients with operable breast cancer to receive pre- or postoperative anthracycline/cyclophosphamide × 4 cycles. With 10 and 16 years of followup, respectively, there was no difference in the primary endpoints of disease-free survival (DFS) or overall survival (OS) in either study [33, 34]. Meta-analyses also confirm equivalence of preoperative and postoperative chemotherapy for endpoints of death (summary RR 1.00, 95% CI = 0.90–1.12), disease progression (summary RR 0.99, 95% CI = 0.91–1.07), and distant disease recurrence (RR 0.94, 95% CI = 0.83–1.06) [35, 36]. In regards to overall antineoplastic effect, these data suggest that survival depends more on intrinsic biologic factors of the primary tumor itself rather than the timing of chemotherapy (before or after surgery).

One potential caveat regarding NCT was raised in these early trials. In NSABP B-18, there was a nonsignificant trend towards increased ipsilateral breast tumor recurrence (IBTR), 13% in the NST arm versus 10% in the adjuvant arm [34]. In the Mauri meta-analysis, NCT was associated with a statistically significant increase in locoregional recurrence (LRR) (RR 1.22, 95% CI = 1.04–1.43), although this was primarily due to inclusion of trials in which patients were treated with NCT and radiation therapy alone, omitting surgery [35]. 

A retrospective analysis of 340 patients treated with NCT at MD Anderson identified an IBTR rate of 5% and LRR rate of 9%, comparable to IBTR/LRR rates with conventional postoperative therapy [37]. Features associated with LRR risk included cN2/N3 disease, lymphovascular space invasion, residual disease >2 cm in size, and a multifocal pattern of residual disease. These features were later incorporated into a prognostic index to help identify patients at higher risk for LRR after NCT [38]. In general, patients with the following features after NCT should not be offered BCT: skin involvement/edema, residual tumor >5 cm, diffuse calcifications, residual multicentric disease, or inability to receive adjuvant radiotherapy.

### 2.3. Increased BCT Rates

Clinical response to NCT correlates with tumor downstaging and increased rates of BCT. In NSABP B-18, there was a statistically significant difference in frequency of BCT for patients in the NCT group (67% versus 60%, *P* = .002) [34]. In EORTC 10902, there was also a statistically significant difference in BCT rates (35% versus 22%) [33]. Interestingly, BCT rates across a number of NCT trials vary dramatically, ranging between 13 and 83%, reflecting differences in inclusion criteria, NCT regimen, patient preference, and surgeon practice. Taken together, these data have established that NCT can allow for BCT in a significant proportion of patients for whom mastectomy was the initially preferred option.

### 2.4. Prognostic Relevance of Response to NCT

Degree of response to NCT positively correlates with overall and disease-free survival. In NSABP B-18, patients who achieved pathologic complete response (pCR) had better OS at 16 years of followup (83.7% versus 55.7%, HR = 0.32, *P* < .00001). Similar results were seen in NSABP B-27, with OS at 8 years favoring those achieving pCR (89.4% versus 73.6%, HR = 0.36, *P* < .00001) [34]. 

Variation in the definition of pCR has limited comparison across studies, prompting the development of an alternative measurement index, residual cancer burden (RCB) [39]. The RCB is derived from measurements of residual primary tumor, cellularity of the tumor bed, and axillary nodal burden, and therefore more accurately reflects the broad spectrum of residual disease encountered in clinical practice. The RCB is grouped into four categories (RCB 0, I, II, III), with RCB 0 being equivalent to pCR and the other three categories representing increasing levels of residual disease. In a validation study, patients with RCB 0 or RCB I had similar DFS, suggesting that near-pCR may be prognostically equivalent to pCR. Moreover, the RCB stratified prognosis within posttreatment AJCC stages (“y” stage), providing valuable prognostic information for progressive levels of residual disease.

### 2.5. Development of Current Standards

As in the adjuvant setting, no “optimal” neoadjuvant chemotherapy regimen has been defined. There are now a number of effective options from which to choose and, in general, consensus guidelines suggest that regimens recommended for use in the adjuvant setting are appropriate for use in the neoadjuvant setting [40]. 

The incorporation of taxanes into NCT results in significant improvement in pCR rates. In NSABP B-27, patients received neoadjuvant AC (4 cycles) and were subsequently randomized to receive no further chemotherapy, neoadjuvant docetaxel (4 cycles), or adjuvant docetaxel (4 cycles). Patients in the neoadjuvant docetaxel arm experienced twice as many pCRs (26% versus 13%, *P* < .001), but no benefit in DFS or OS was demonstrated [34]. The Geparduo trial confirmed that sequential administration of anthracycline and taxane is more effective than concurrent administration [41]. 

For patients with Her2-positive disease, the addition of trastuzumab to standard NCT results in dramatic improvement in pCR and DFS rates. Buzdar et al. reported an increase in pCR rate from 26.3% to 65.2% (*P* = .016) in patients receiving NCT versus NCT plus trastuzumab [42]. Similar results were seen in the NOAH trial, with improved pCR rate (23% versus 43%, *P* = .002) and DFS rate (53.5% versus 70.1%, *P* = .007) with addition of trastuzumab to NCT [43]. Rates of clinically significant cardiac dysfunction were low in both studies. ACOSOG Z1041, currently accruing, will definitively address the question of safety in regards to coadministration of epirubicin with trastuzumab. 

In general, choice of NCT regimen is determined largely by tumor characteristics, patient factors, physician preference, and institutional/regional trends. At MD Anderson, for Her2-negative tumors, the standard NCT regimen is weekly paclitaxel (12 doses) followed by FAC (4 cycles). For Her2-positive tumors, the standard NCT regimen is weekly paclitaxel-trastuzumab (12 doses) followed by FEC-trastuzumab (4 cycles), with herceptin completed to 1-year total postoperatively.

### 2.6. Modifying NCT Based on Response

One hypothetical benefit of NST is the ability to assess *in vivo* response and adjust therapy to maximize response. Smith et al. examined this issue by treating patients with 4 cycles of neoadjuvant anthacycline-based chemotherapy. Responders were then randomized to receive an additional 4 cycles of anthracycline or 4 cycles of docetaxel, and nonresponders all received 4 cycles of docetaxel. Interestingly, responders preferentially benefitted from incorporation of docetaxel (pCR rate 30.8% versus 15.4% in anthracycline-only group, *P* = 0.04), whereas nonresponders derived essentially no benefit (pCR rate 1.8%) [44].

Similar findings were identified in the GEPARTRIO trial, where patients received two cycles of neoadjuvant TAC (docetaxel, doxorubicin, cyclophosphamide), with nonresponders subsequently randomized to 4 additional cycles of TAC versus 4 cycles of vinorelbine/capecitabine. No difference was identified in the pCR rate (5.3% versus 6.0%) [45].

Taken together, these data suggest that clinical nonresponders may harbor tumors that are broadly chemoresistant. Further studies are needed to identify predictors of chemoresistance as well as novel agents to reestablish response in this population.

### 2.7. Predictors of NST Response

Tumor histology may be an important predictor of NST response. Across multiple studies, pCR rates for invasive lobular carcinomas (ILC) are in the 1–3% range, as opposed to pCR rates in the 15% range for invasive ductal carcinomas (no special type) [46]. Comparing HR-positive ILC and IDC, pCR rates for ILC remain relatively low (4% versus 9%, *P* = .03). Moreover, BCS rates after NCT with ILC are lower than for IDC (16% versus 29%, *P* = .003) [47]. Some authors have suggested that ILC should not be treated with NCT [48], and this remains an area of investigation.

ER/PR status independently predicts likelihood of achieving pCR. In the ECTO trial, pCR rates were significantly lower for ER-positive versus ER-negative tumors (12% versus 42%, *P* < .0001) [49]. A larger retrospective analysis from MD Anderson supports the conclusion that HR-positive tumors have lower pCR rates (8% versus 24%, *P* < .0001) [50]. Importantly, although the odds of achieving pCR are lower in HR-positive patients, the OS and PFS benefit of achieving pCR is retained. Tumor differentiation and proliferation rate also correlates with response in a similar fashion; favorable biologic markers such as lower grade and low proliferation rate predict for lower pCR rates. An integrated view of the histologic and molecular predictors, including Her2/neu status, is provided by the Sorlie classification (Table 2) [51]. 

While the majority of data has explored factors predictive of tumor response to NST, fewer studies have examined predictors of disease progression during NST. A recent retrospective study from MD Anderson identified progressive disease in only 3% of patients receiving neoadjuvant chemotherapy. In these patients, multivariate analysis identified African-American race, higher T stage, and ER negativity as predictors of progression, with high tumor grade, high Ki-67 score, and PR negativity also identified on univariate analysis [52]. Interestingly, some of these factors (HR negativity, high grade, high Ki-67) also predict for higher pCR rates, suggesting that such tumors may represent two pathologically similar but clinically distinct subpopulations. 

Work continues on molecular profiling assays to further characterize tumor subtypes and identify patients most likely to derive benefit from NST.

### 2.8. Neoadjuvant Endocrine Therapy

HR-positive disease comprises approximately 75% of breast cancer cases. Two findings propelled interest in neoadjuvant endocrine therapy: (1) the efficacy of endocrine therapy in the adjuvant setting and (2) lower pCR rates with neoadjuvant chemotherapy in HR-positive, Her2-negative tumors. 

In the past, neoadjuvant endocrine therapy was studied primarily in older, frailer patients with HR-positive tumors. More recently, three major studies established the utility of neoadjuvant endocrine therapy in downstaging HR-positive tumors and improving BCT rates. P024 randomized postmenopausal women to letrozole or tamoxifen ×4 months prior to surgery. Achievement of the primary endpoint (clinical response by palpation) favored the letrozole arm (55% versus 36%, *P* < .001), as did rate of BCS (45% versus 35%, *P* = .002) [53]. Two other phase III trials demonstrated trends favoring clinical response with anastrozole over tamoxifen, and confirmed higher rates of BCT with anastrozole [54, 55]. 

Two published studies have compared neoadjuvant chemotherapy with neoadjuvant endocrine therapy. These were smaller trials but confirmed that clinical response rates were generally similar across groups, with the notable exception of premenopausal patients, who experienced lower response rates with neoadjuvant endocrine therapy [56, 57]. An important feature across neoadjuvant endocrine trials is that pCR rates are generally low (1–5%). Other markers, such as Ki-67, may correlate better with long-term outcome and have led to the creation of a novel index that combines pre- and posttreatment tumor features intended to predict recurrence risk and need for adjuvant chemotherapy [58]. 

Currently, neoadjuvant endocrine therapy is not recommended in premenopausal women. In postmenopausal women with HR-positive, Her2-negative tumors, use of an neoadjuvant aromatase inhibitor can be considered on an individualized basis.

### 2.9. Future Directions

Breast conservation is an important goal for many patients with operable and locally advanced breast cancer. Recent advances in NST highlight the importance of a tailored approach based on tumor and patient characteristics. Developing novel approaches to individualize NST and treat chemoresistant disease is likely to lead to higher BCT rates and improved outcomes.

## 3. Radiation Oncology Perspective

There have been significant advances in radiation therapy as an adjuvant for breast conserving surgery in the last several years. The advances have been in both whole-breast irradiation and partial-breast irradiation. The improvements have spanned from computer tomography (CT) imaging for target definition to more advanced radiation treatment planning software resulting in more homogeneous dose distributions. The end result is less acute and late treatment toxicity with improved cosmesis and at least equal treatment efficacy compared to previous methods of radiation planning and treatment delivery.

### 3.1. Whole-Breast Radiation

Adjuvant radiation therapy after breast conserving surgery (BCS) is recommended to eradicate potential microscopic residual disease adjacent to the original tumor site after segmental resection, which may be present 30 to 40% of the time [60]. The majority of in-breast recurrences after BCS are in the same quadrant as the original primary tumor [61–63]. Therefore, it is important to ensure adequate coverage of the remaining breast tissue adjacent to the surgical bed. This can be achieved quite well with whole-breast irradiation. The advent of computed tomography (CT) simulation has allowed us to contour the surgical lumpectomy cavity as well as visualize normal structures such as heart and lung. 

 In the supine position, the patient can be positioned comfortably on an angle board at a set angle in a custom-designed cradle to reproduce her treatment position each day. The ipsilateral arm is abducted and rotated above the head, to keep it out of the radiation fields. The semireclined position tends to be more comfortable for the patient, minimizing the risk of patient motion during treatment. The angle board can be fixed to the treatment table to ensure exact patient positioning during treatment. A cradle is then made, customized to the patient and her position to maintain the patient's upper body position in the same location each day. The custom-made cradle is fixed to the angle board. In the CT simulator, radio-opaque wire can be placed to localize the surgical lumpectomy scar as well as to define preliminary field borders. Then CT images at 2.5 mm to 3 mm slice thickness are obtained through the breast. With a large-bore CT simulator, even patients who require bilateral breast irradiation due to bilateral synchronous breast cancer can be imaged with a single CT data set. 

The CT data set is then transferred to 3D CT-based treatment planning system. This allows the patient to be discharged from the clinic, while the dosimetrist and radiation oncologist can optimize the treatment for the individual patient. The surgical cavity is outlined as well as the heart, if treatment is to the left breast. Often times, the surgeon has left surgical clips outlining the edges of the lumpectomy cavity, further aiding in the targeting of the surgical bed. Field gantry angles, collimation, beam weightings, and energies are chosen to allow a homogenous dose distribution throughout the breast while minimizing “hot” and “cold” regions of dose. In addition, the volume of lung included in the radiation treatment field is evaluated. In general, only a limited volume of lung is encompassed in the tangential fields, just enough to ensure coverage of the deep breast tissue toward the chest wall and accounting for breathing during treatment, resulting in some breast movement. With 3D visualization of the operative bed and the rest of the breast, the medial and lateral tangential beams can be custom-designed to ensure dosimetric coverage of the targeted surgical bed and/or surgical clips and surrounding breast tissue while allowing for potential daily setup errors and patient motion during treatment.

Standard whole breast irradiation is 45–50 Gy delivered at 1.8–2.0 Gy/fraction over 4.5–5 weeks followed by a boost dose to the surgical bed and scar of 10–16 Gy delivered at 2 Gy/fraction over 1–1.5 weeks. Total dose is 60–66 Gy over a course of 6–6.5 weeks. There is now also interest in a hypofractionated course of whole breast irradiation, delivering the treatment over 3–4 weeks, rather than over 6–6.5 weeks. A randomized study by Whelan et al., reported an equivalent outcome for tumor control and normal tissue effects at 10 years. In an exploratory subgroup analysis, patients with high-grade tumors treated with the hypofractionated regimen had a higher incidence of local recurrence than those treated with the standard fractionation whole-breast irradiation regimen [64]. At The University of Texas MD Anderson Cancer Center, we currently reserve the use of the hypofractionated whole-breast irradiation regimen of 42.4 Gy at 2.65 Gy/fraction to postmenopausal women with pT1, N0, M0 invasive breast cancer of low to intermediate grade that is estrogen receptor positive. We often follow this with a boost dose of 10 Gy in four fractions to the surgical bed and scar.

**Figure 3:**
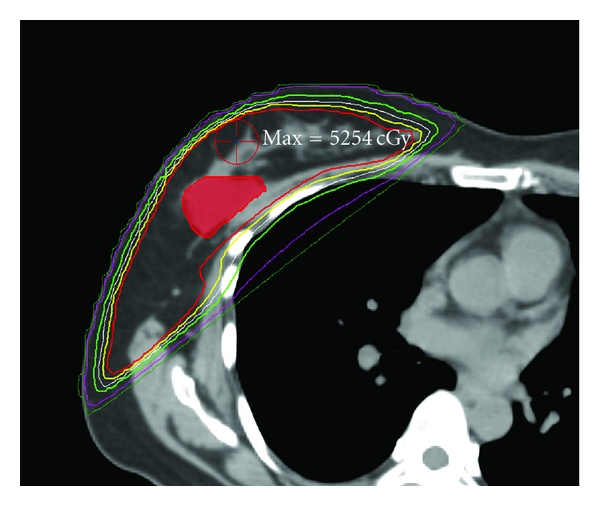
An example of a step and shoot forward-planned IMRT breast treatment plan. Notice the very homogenous coverage of the breast by the 5000 cGy isodose line in red, just skimming at the edge of the pectoralis muscle, yet limiting volume of lung irradiated.

The use of 3D CT-based treatment planning has improved the dosimetric homogeneity and visualization of dose exponentially over 2D treatment planning. The prescription dose is specified to an isodose line, generally following along the pectoralis muscle surface and encompassing the breast tissue. Given the 3D CT data set, we are able to evaluate the dose deposited in every area of the breast, not just in a single plane at the midlevel of the breast, as was previously the case. In addition, dose calculations now take into account the heterogeneity of the various tissues included within the treatment field, such as the breast parenchyma, rib, and lung. We are able to optimize the tangential whole breast plans, so that significant volumes of breast tissue do not exceed 105% of the prescribed dose. This has been made possible by sophisticated treatment planning software. The “field-in-field” technique, also known as “field-in-field forward-planned intensity modulated radiotherapy (FiF-IMRT)” provides excellent dose homogeneity in all three dimensions of the breast, as compared to plans generated in the 2D era with wedges. This improved dose homogeneity also allows for better dosimetric coverage of the breast and lumpectomy cavity. The improved homogeneity reduces increased dose deposits (“hot spots”) such as in the inframammary fold, axilla, and narrow portion of the breast toward the nipple/areolar complex. The end result is less acute and long-term side effects of radiation therapy, such as acute dermatitis, edema, hyperpigmentation, and chronic breast edema [65]. Using this technique, one starts with an open tangential beam arrangement. High-dose volumes (e.g., 115%, 110%, 105%) are sequentially blocked with custom multileaf collimation, generating smaller field segments within the main medial and lateral open tangential fields. These smaller field segments can be delivered as separate treatment fields (field in field) or as part of the original fields with a step-and-shoot technique. A percentage of the overall planned dose each day is delivered with these smaller fields within the main open field (Figure 3). In addition, the scattered dose to the contralateral breast with the FiF-IMRT technique is less than the dose delivered with a conventional wedge technique [66]. The FiF-IMRT technique, the step-and-shoot forward-planned IMRT technique, and an inversely-planned breast IMRT technique can all improve dose homogeneity compared with a conventional wedge technique. For patients with larger breast sizes and/or separation distances, higher energy photons may be a necessary component of the IMRT treatment planning, to ensure adequate dose homogeneity.

**Figure 4:**
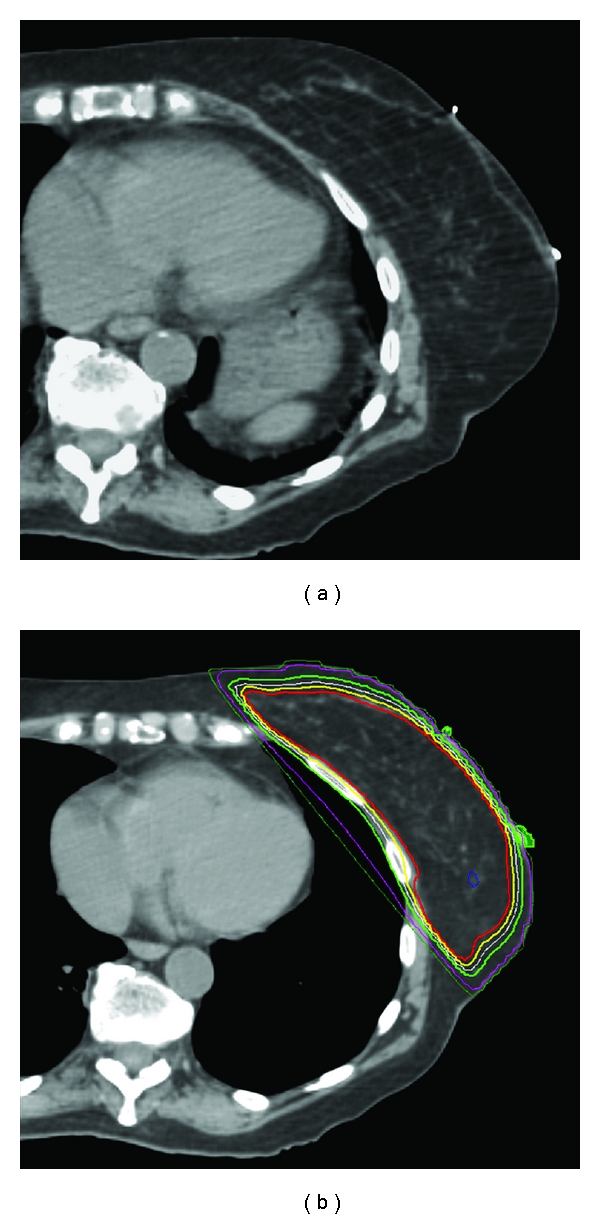
Deep inspiration breath-hold technique: notice how with deep inspiration the cardiac shadow has moved away from the chest wall, allowing the radiation beams to adequately cover the breast and avoid direct irradiation of the heart.

To prevent radiation-induced heart disease resulting in possible death 15 or more years after the completion of breast cancer treatment, there is now significant effort made to avoid treatment of the heart after BCS [67]. There are various methods in the 3D era of radiation treatment planning to avoid direct cardiac irradiation. The anterior lateral aspect of the heart, including the left anterior descending artery, can be very close to the posterior edge of the tangential fields if treating the left breast. In some cases, changing the gantry angle, the collimator angle or the posterior field border can exclude the heart from the radiation fields while still providing coverage of the targeted segmental resection site. In other cases, small cardiac blocks utilizing custom-designed multileaf collimation can be placed to avoid cardiac irradiation. In situations where the heart cannot be avoided due to its proximity to the chest wall and surgical bed in the free-breathing setting, a deep inspiration breath-hold technique can be utilized. Planning is performed on CT images obtained during the inspiratory phase of the respiratory cycle, which displaces the heart both inferiorly and medially, away from the left chest wall, and thus away from the tangential beams of radiation [68] (Figure 4).

**Figure 5:**
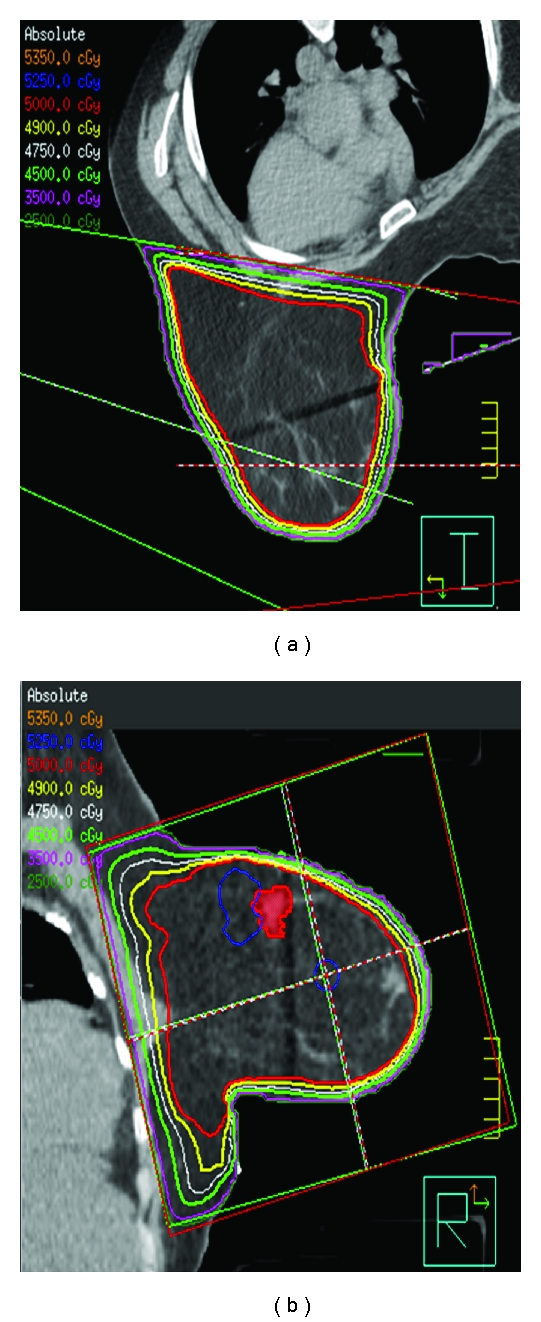
Prone breast treatment positioning: notice the avoidance of the heart and the rib cage in the treatment fields. The prone position has avoided the folding of the breast onto the upper abdominal skin, and thus reduced the dose delivered in the inframammary fold.

Prone positioning of the patient is another technique that may be used in whole-breast irradiation. There are several occasions where it may serve the patient better than supine positioning. In general, the prone position allows the breast and surgical bed to fall away from the chest wall. As a result, more normal tissue can be excluded from the tangential treatment fields, such as the heart, lung, and in some cases the rib cage. Because of the more limited posterior coverage dosimetrically in the prone position, surgical beds that lie close to the chest wall may not be optimally treated in this position. Another advantage of the prone position is to optimize treatment positing of a pendulous breast. The pendulous breast hangs down above the treatment table, thus avoiding skin folding in the inframammary fold and axilla resulting in increased acute skin reactions. The width of the breast is also reduced, reducing the beam energy necessary for treatment and improving the homogeneity of the treatment plan (Figure 5).

### 3.2. Accelerated Partial Breast Irradiation

**Figure 6:**
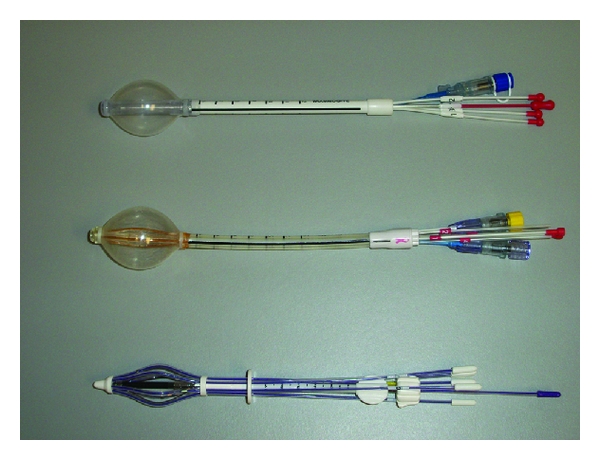
Single-entry hybrid breast brachytherapy catheters. Top: MammoSite ML, middle: Contura MLB 4.0–5.0 cm, and bottom: SAVI 6-1 Mini.

Accelerated partial breast irradiation (APBI) is attracting more attention from both physicians and patients as an alternative to adjuvant whole-breast irradiation in early stage breast cancer as the efficacy data continues to mature, suggesting similar in-breast recurrence rates as whole breast irradiation [69–71]. APBI targets the breast tissue immediately surrounding the lumpectomy cavity; the breast tissue at highest risk of harboring residual cancer cells. As a result, more normal tissue, such as the rest of the breast tissue, rib cage, heart, and lung is spared. The oldest technique of accelerated partial breast irradiation is multicatheter interstitial brachytherapy. Approximately 15 to 20 catheters are laid within and immediately surrounding the segmental resection cavity, treating approximately a 1.5 cm margin of breast tissue away from the edge of the cavity. When delivered with a high dose rate iridium-192 source, a commonly used total dose and fractionation schedule is 34 Gy at 3.4 Gy/fraction delivered twice a day, six hours or greater apart for 10 fractions. There is up to 10-year efficacy and cosmetic data with the multicatheter interstitial technique, some of which was delivered as a low dose rate implant, noting low recurrence rates in the breast, in general 5% or less, and similar to whole-breast irradiation [71, 72]. Multicatheter interstitial brachytherapy required expertise in needle placement, and not all patients embraced the idea of multiple needles placed through the breast. As a result, APBI with a brachytherapy technique did not blossom until FDA approval of the MammoSite device in 2002. A similar volume of breast tissue is treated as compared to multicatheter interstitial brachytherapy when treating to a volume 1 cm around the periphery of the MammoSite balloon when expanded within the lumpectomy cavity [73, 74]. As five-year data has been published in well-selected patients, again demonstrating low recurrence rates in the breast treated with adjuvant APBI with a MammoSite balloon applicator [69], interest emerged in making APBI available to more women. The MammoSite balloon's simplicity of a single lumen device with a single entry into the breast, as compared to multicatheter brachytherapy, was also its limitation. Women with smaller breast sizes and/or lumpectomy cavities that approached too close to skin, within 5 mm, were not able to be treated with a MammoSite balloon, even if the tumor pathology and age of the patient were compatible with APBI. We have now seen come to market three other single-entry hybrid breast brachytherapy devices that meld the simplicity of just one entry site for the device, with the dosimetric versatility of the multiple catheters in the ability to pull dose away from skin and/or rib. The SAVI (Strut-Adjusted Volume Implant), the Contura MLB (Multi-Lumen Balloon), and the MammoSite ML (Multiple Lumen) all offer a dosimetric improvement in control over the original MammoSite (Figure 6). The Contura MLB has four catheters offset 5.5 mm from the central catheter, totaling five catheters. The balloon comes in two sizes, 4.0–5.0 cm and 4.5–6.0 cm. At lower fill volumes, the Contura is somewhat elliptical in shape. At higher fill volumes, the balloon is more spherical. There are two vacuum ports located at the distal and proximal ends of the balloon. The vacuum port allows air and/or seroma to be aspirated, that may have been separating the targeted breast tissue away from the balloon. The MammoSite ML has three catheters surrounding a central catheter, resulting in a total of four catheters. The triad of catheters are offset 3 mm from the central lumen. The diameter of the balloon is 4.0–5.0 cm. It is variable inflatable, similar to the Contura MLB. The single lumen MammoSite balloons are also available in diameters of spherical 4.0–5.0 cm, spherical 5.0–6.0 cm, and an ellipsoidal balloon of 4.5–6.0 cm. The SAVI device is a multilumen single-entry breast brachytherapy device without a balloon. The device comes in four sizes, ranging from the 6-1 Mini SAVI to the 10-1 SAVI. The 6-1 Mini measures 2.4 cm × 5 cm. The SAVI 10-1 device measures 5 cm × 7.5 cm. The numbers represent the number of catheters surrounding the central catheter. Therefore, the 6-1 Mini has 6 catheters surrounding a central catheter. The 10-1 SAVI has 10 catheters surrounding a central catheter. Additional sizes include the SAVI 6-1 and the SAVI 8-1. The benefit of these multiple breast brachytherapy device types and sizes is more women who are candidates for APBI based on age and disease pathology will be able to receive APBI with a single-entry device if they chose. The limitations of distance of lumpectomy cavity to skin surface and/or rib surface have been significantly reduced with these multilumen catheters. The ability to maximize conformance of the device to the lumpectomy cavity edge in all dimensions has also been improved by the various sizes and shapes of the devices, and in the case of the Contura MLB, by the vacuum ports.

**Figure 7:**
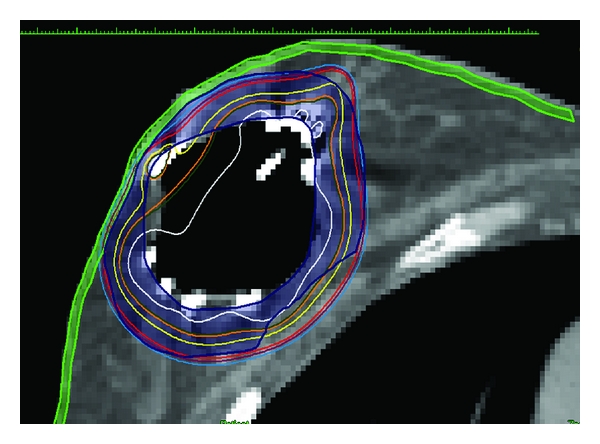
SAVI 10-1 implant. Notice how the isodose lines pull away from the skin surface. The maximum dose to a 0.1 cc volume of skin is 101% of the prescribed 34 Gy. The maximum dose to a 0.1 cc volume of rib is 100% of the prescribed 34 Gy. There is excellent dosimetric coverage of the PTV_EVAL (V95 = 96% with only 0.9% nonconformance).

To maximize the success of placement of the single-entry brachytherapy device with excellent conformance, we performed a limited noncontrast CT in our CT simulator up to two days prior to planned placement of the device. Then 2D, 3D, and serial axial images may be sent electronically to the breast surgeon, with discussion of cavity dimensions, cavity volume, and minimum distance of the lumpectomy cavity edge to skin surface and rib surface. The radiation oncologist and breast surgeon can decide jointly which device and size of device appears optimal for the individual patient. The patient is then simulated for final brachytherapy treatment planning one to two days after placement of the device. Given the dosimetric versatility of the multi-lumen devices, we strive for V95 greater than or equal to 95%, meaning 95% of the prescription dose is covering greater than or equal to 95% of the planning target volume for evaluation (PTV_EVAL). The PTV_EVAL is defined as the volume encompassed by a 1 cm expansion around the periphery of the device, limited to 5 mm from skin surface in the case of a balloon device, and 2 mm from skin surface in the case of the SAVI, and limited by posterior breast tissue, while subtracting out the lumpectomy cavity volume). This coverage should take into account any nonconformance of the device to breast tissue. In addition, we are able to routinely limit the dose to skin, defined as a 2 mm rind of tissue from skin surface into breast tissue) to under 115% of dose to a 0.1 cc volume, and often much lower. This is a great improvement over the original limitation of a 145% of prescribed dose to skin surface from the original single lumen MammoSite [75]. In a similar fashion, we are able to limit dose to the rib cage as well. As a result, we expect acute and late toxicity of APBI with a single-entry device to further improve even as we treat lumpectomy cavities that were previously excluded due to proximity to rib or skin (Figure 7).

Spacers have also been developed to be used at the time of surgery at the discretion of the operating surgeon to establish the tract and hold the cavity shape. After confirmation that the final pathology is compatible for APBI, the spacer can be removed and the final catheter placed in the clinic. The spacer alleviates the need for establishing the tract in the clinic.

There are other noninvasive methods of APBI undergoing evaluation. They include 3D conformal radiotherapy (3D-CRT), intensity-modulated radiotherapy (IMRT), volumetric-modulated arc therapy (VMAT), and continuous arc rotation of the couch (C-ARC) [76]. AccuBoost is a noninvasive breast brachytherapy system which uses mammographic localization and immobilization to direct iridium-192 emissions along orthogonal axes [77]. APBI utilizing multiple proton beams is also under investigation [78].

It is an exciting time in breast radiation oncology, as with advancing technology we strive with improved accuracy to treat the targeted tissue potentially harboring residual microscopic cancer cells, while minimizing dose to normal tissues.

## 4. Conclusion

There is no question for those that treat breast cancer patients that a significant proportion of time goes into counselling the patient and family in terms of the various options available to the patient for surgery and adjuvant treatments. The practitioner should present all the options medically relevant to the specific patient's presentation and guide the patient through the decision-making process so that her final decision best suits her circumstances and comfort level. The data and technologic advances for optimization of breast conservation are developing at lightning speed and patient driven care and demands will likely continue the push for state-of-art treatment, which will only contribute to improved care of the breast cancer patient.

## Figures and Tables

**Table 1:** Hypothetical advantages/disadvantages of NST. Only one hypothesis (in bold) is corroborated by current data.

Advantages	Disadvantages
Delivery of systemic therapy via intact tumor vasculature	Inaccurate post-NST staging
*In vivo* response assessment with potential to tailor therapy accordingly	Treatment of larger overall tumor burden
Earlier treatment of micrometastatic disease	Delay in curative local therapy
**Potential to downstage and facilitate BCT**	Increased risk for surgical/radiation complications

**Table 2:** Sorlie molecular subtypes and response to neoadjuvant chemotherapy [50, 51, 59].

Sorlie subtype	Receptor correlates	Estimated pCR rates
Luminal A	HR+, Her2−	7–12%
Luminal B	HR+, Her2+	15–25%
Her2-like	HR−, Her2+	20–25% (without Herceptin) 40–60% (with Herceptin)
Basal	HR−, Her2−	25–55%
